# Development of a Recombinase Polymerase Amplification Assay for Rapid Detection of the *Mycobacterium avium* subsp. *paratuberculosis*

**DOI:** 10.1371/journal.pone.0168733

**Published:** 2016-12-19

**Authors:** Sören Hansen, Jenny Schäfer, Kim Fechner, Claus-Peter Czerny, Ahmed Abd El Wahed

**Affiliations:** Division of Microbiology and Animal Hygiene, Institute of Veterinary Medicine, Department of Animal Sciences, Faculty of Agricultural Sciences, Georg-August University, Goettingen, Germany; University of Helsinki, FINLAND

## Abstract

**Background:**

The detection of *Mycobacterium avium* subsp. *paratuberculosis* (MAP) infections in ruminants is crucial to control spread among animals and to humans. Cultivation of MAP is seen as the gold standard for detection, although it is very time consuming and labour intensive. In addition, several PCR assays have been developed to detect MAP in around 90 minutes, but these assays required highly sophisticated equipment as well as lengthy and complicated procedure.

**Methodology/Principal Findings:**

In this study, we have developed a rapid assay for the detection of MAP based on the recombinase polymerase amplification (RPA) assay targeting a MAP specific region, the IS900 gene. The detection limit was 16 DNA molecules in 15 minutes as determined by the probit analysis on eight runs of the plasmid standard. Cross reactivity with other mycobacterial and environmentally associated bacterial strains was not observed. The clinical performance of the MAP RPA assay was tested using 48 MAP-positive and 20 MAP-negative blood, sperm, faecal and tissue samples. All results were compared with reads of a highly sensitive real-time PCR assay. The specificity of the MAP RPA assay was 100%, while the sensitivity was 89.5%.

**Conclusions/Significance:**

The RPA assay is quicker and much easier to handle than real-time PCR. All RPA reagents were cold-chain independent. Moreover, combining RPA assay with a simple extraction protocol will maximize its use at point of need for rapid detection of MAP.

## Introduction

Paratuberculosis (Johne's disease) is caused by the Gram-positive, aerobic, non-motile, non-spore-forming and acid fast *Mycobacterium avium* subsp. *paratuberculosis* (MAP) [1]. The disease is characterized by a chronic progressive course marked by emaciation and accompanying lethal enteritis. MAP infects primarily large and small ruminants leading to diarrhea, weight loss and decreased milk production [2]. Particularly, animals infected subclinically represent a hotspot for transmitting MAP within the herd [3]. In humans, MAP was isolated from patients with Crohn's disease (inflammatory bowel disease) [4–6] and for more than 100 years, MAP has been discussed to be the causative agent [7]. Johne's disease is leading to huge economic losses in dairy production and since no pharmacological treatment or a licensed vaccine are available, early detection of the source of a MAP infection in a herd is very crucial to diminish such losses.

The gold standard for the diagnosis of MAP is culturing the bacteria [8], which takes up to 12 weeks and is only possible in highly equipped laboratories. Direct detection of MAP antibody with ELISA has been extensively applied but the clinical sensitivity and specificity is lower than the molecular assays [8]. Many real-time, conventional, semi-nested and nested PCR assays have been developed for the detection of MAP in up to 3 hours [9–11]. Nevertheless, a big challenge is the implementation of PCR in mobile point of need systems, because of its rapid thermocycling between the denaturation temperature, 95°C, and approximately 50°C for primer annealing together with a precise temperature control [12].

Unlike PCR, isothermal DNA amplification assays do not need a controlled thermal cycling complex device. Moreover, these assays offer greater utility in the field by including simplistic reactor designs or portable heat sources [13]. Recombinase polymerase amplification (RPA) is an isothermal technology, which amplifies DNA at a constant temperature between 25°C and 42°C [13]. RPA depends on a combination of recombinase, single strand binding protein and strand displacing DNA polymerase for the DNA amplification step. Real-time detection of RPA amplicons relies on the exo-nuclease enzyme, which cuts at the basic mimic site presence between fluorophore and quencher in the RPA exo-probe. The RPA is extremely fast (3–15 minutes) and all reagents are cold chain independent. There have been developed several RPA assays to detect a wide range of different pathogens such as group B streptococci, *Brucella* or *Mycobacterium tuberculosis* [13–15].

In this study, a real-time RPA assay detecting MAP-DNA was developed. The assay sensitivity, specificity and cross reactivity were determined. The clinical performance of the MAP RPA assay was evaluated by 48 MAP-positive and 20 MAP-negative blood, sperm, faecal and tissue samples. All results were compared by a well-established real-time PCR [16].

## Materials and Methods

### Ethical statement

In total, the study included 68 archived DNA samples, which have been collected during routine veterinary examination in the Institute of Veterinary Medicine, Goettingen. All samples were taken under consideration of the German codex “Gute Veterinärmedizinische Praxis”.

### Generation of a molecular and genomic DNA MAP Standards by PCR

MAP (ATCC 19698) was ordered from Leibniz Institute DSMZ-German Collection of Microorganisms and cell cultures (DSMZ, Braunschweig, Germany, ID: 44133). DNA was extracted as follows: after culturing MAP on Herrold´s Egg Yolk Agar Slants medium containing mycobactin and amphotericin B, nalidixinacid, vancomycin (BD, Franklin Lakes, NJ, USA) for 5 weeks, pure colonies were suspended in 100 μl water and incubated at 99°C to 100°C with shaking at 300 rpm in a Bioer Mixing Block MB-102 (Bioer Technology, Hangzhou, China). After 20 minutes, the suspension was centrifuged at 14000 g for 10 minutes. Then the supernatant was collected and centrifuged at 14000 g for 10 minutes. The amount of DNA in the supernatant was measured by a NanoDrop ND-1000 spectrometer (Thermo Scientific, Waltham, MA, USA). A genomic DNA standard containing 10 ng/μl to 10 fg/μl of DNA dissolved in water was prepared to test the analytical sensitivity of the RPA assay. To generate the plasmid standard, a 587 bp fragment was amplified covering the nucleotides 183 to 769 of MAP Gene IS900 sequence (Genbank accession number: AF416985.1) using the published primer sequences 5´-GTCGGCGTGGTCGTCTGCTGGGTTGAT-3 as a forward primer (FP) and 5´-GCGCGGCACGGCTCTTGTTGTAGTC-3 as a reverse primer (RP) [17]. The PCR reaction was performed on a T3000 thermocycler (Biometra GmbH, Goettingen, Germany) using the following reaction mix: 11.8 μl molecular biology H_2_O, 2 μl BSA (Carl Roth GmbH + Co. KG, Karlsruhe, Germany), 2 μl 10x Standard Reaction Buffer containing MgCl2 (Biotools B&M Labs. S.A.,Madrid, Spain), 0.4 μl DMSO (Carl Roth GmbH + Co. KG, Karlsruhe, Germany), 0.4 μl of each primer (10 μM stock solution), 0.1 μl Biotools DNA polymerase (Biotools B&M Labs. S.A.,Madrid, Spain), 0.1 μl of each of the 10 μM aNTP, tNTP, cNTP and gNTP stock solutions (Carl Roth GmbH + Co. KG, Karlsruhe, Germany) and 2.5 μl of the template. The PCR cycles were programmed as follows: initial denaturation step at 95°C for three minutes, then thirty cycles of 95°C/30 sec, 64°C/60 sec and 72°C/60 sec and final extension step at 72°C for eight minutes. The amplified fragment was then cloned using the pGEM-T Easy Vector Systems I kit (Promega, Madison, USA) according the manufacture instructions. The number of plasmids were calculated using an equation as previously described [18]. The plasmids were linearized using the Ncol-HF (New England Biolabs, Ipswich, MA, USA).

A dilution range of 10^0^ to 10^6^ molecules/μl of the standard was prepared. Both the molecular plasmid and genomic DNA standard were tested using a published quantitative real-time PCR protocol ([16], patent number: EP2841596A1) as described below.

### Real-time PCR

The real-time PCR assay was performed on a LightCycler 480 (Roche, Mannheim, Germany) using the LightCycler 480 Probes Master Kit (Roche, Mannheim, Germany) to amplify a 139 bp long fragment (nucleotides 523 to 636 of the Genbank accession number: AF416985.1). The Reaction volume contained 10 μl Light Cycler 480 Probes Master mix, 0.5 μl of each 10 μM FP: 5´-TACCGCGGCGAAGGCAAGAC-3´ and RP: 5´-CGGAACGTCGGCTGGTCAGG-3´, 1 μl of 10 μM probe: 5´-FAM-ATGACATCGCAGTCGAGCTG-BHQ-1-3 and 3 μl molecular biology H_2_O as well as 5 μl of the DNA template. The LightCycler was programmed as follow: first ten minutes pre-incubation at 95°C, then 45 cycles of 95°C/15 sec, 60°C/30 sec and 72°C/35 sec followed by a final cooling step at 40°C for 30 sec.

### Real-time RPA primers and exo-probes

Three regions (RPA1, RPA2 and RPA3) flanking the IS900 gene sequence were tested to select an area generating the highest RPA assay sensitivity (S1 Fig). In total, 18 forward primers, 25 reverse primers and three exo-probes were tested. The exo-probe was produced by TIB MOLBIOL (Berlin,Germany), while all the primers were purchased from Eurofins MWG Synthesis GmbH (Ebersberg, Germany). RPA primers were purified by gel filtration and the exo-probe by HPLC.

### RPA assay conditions

The MAP RPA assay was performed using the TwistAmp Exo “Improved Formulation” kit (TwistDx Ltd, Cambridge, UK) according to the manuals instruction. Briefly, 29.5 μl Rehydration buffer, 6.7 μl H2O, 2.1 μl of 10 μM of both FP and RP, 0.6 μl of the 10 μM of the exo-probe and 5 μl of the DNA template were added to a freeze dried reaction pellet. For the negative control water was used instead of the DNA template. The RPA reactions were incubated at 42°C for fifteen minutes using a Tubescanner (Twista, TwistDx Ltd, Cambridge, UK). A mixing step by vortexing was performed after 230 seconds of the incubation in order to improve the sensitivity of the assay. The FAM fluorescence signal intensities were visualized on the Tubescanner studio software (version 2.07.06, QIAGEN Lake Constance GmbH, Stockach, Germany). For signal interpretation, threshold and first derivative analysis were applied.

### Analytical sensitivity and specificity

To determine the analytical sensitivity of the RPA assay, the molecular standard (10^6^ to 10^0^ molecules/μl) was tested eight times and the genomic DNA standard (10 fg/μl to 10 ng/μl) in triplicate in both RPA and real-time PCR. The threshold times and the CT values were plotted against the number of molecules detected. Semi-log non-regression analysis was calculated with PRISM (Graphpad Software Inc., San Diego, California) and probit analysis was performed by STATISTICA (StatSoft, Hamburg, Germany). The analytical specificity of the RPA assay was tested with DNA isolated from different bacteria strains listed in Table 1. All bacteria species except *Mycobacterium bovis* were provided by DSMZ and grown on media following the instructions given by the DSMZ. All mycobacteria were tested positive with 16S ribosomal DNA PCR assay to ensure DNA extraction quality [19]. *Mycobacterium bovis* DNA (ATCC: 27289) was provided by Institute for molecular Pathogenesis, Friedrich Loeffler Institut, Federal Research Institute for Animal Health, Jena, Germany.

**Table 1:** List of bacteria species and strains used for determining the cross reactivity of the RPA assay.

Name of Bacteria	DSMZ ID
*Mycobacterium bovis*	N/A
*Mycobacterium avium* subsp. *avium*	44156
*Mycobacterium kansii*	44162
*Mycobacterium marinum*	44344
*Mycobacterium fortuitum*	46621
*Mycobacterium avium* subsp. *Silvaticum*	44175
*Mycobacterium avium intracellulare*	43223
*Mycobacterium phlei*	43239
*Mycobacterium gordonae*	43213
*Mycobacterium smegmatis*	43756
*Mycobacterium scrofulaceum*	43992
*Rhodoccus hoagie*	20307
*Escherichia coli*	30083
*Clostridium perfringens*	756
*Staphylococcus aureus*	799
*Listeria monocytogenes*	15675
*Streptococcus uberis*	20569
*Streptococcus agalactiae*	2134
*Enterococcus faecalis*	1103

### Clinical samples

Archived DNA of MAP positive blood (n = 14), sperm (n = 18), faecal (n = 12) and tissue (n = 4) samples as well as 20 MAP-negative faecal samples were tested in both RPA and real-time PCR in triplicate as described above. DNA extraction was performed by QIAamp DNA Blood Mini Kit (QIAgen GmbH, Hilden, Germany) using the recommended modifications as described previously [16].

## Results

**Fig 1:**
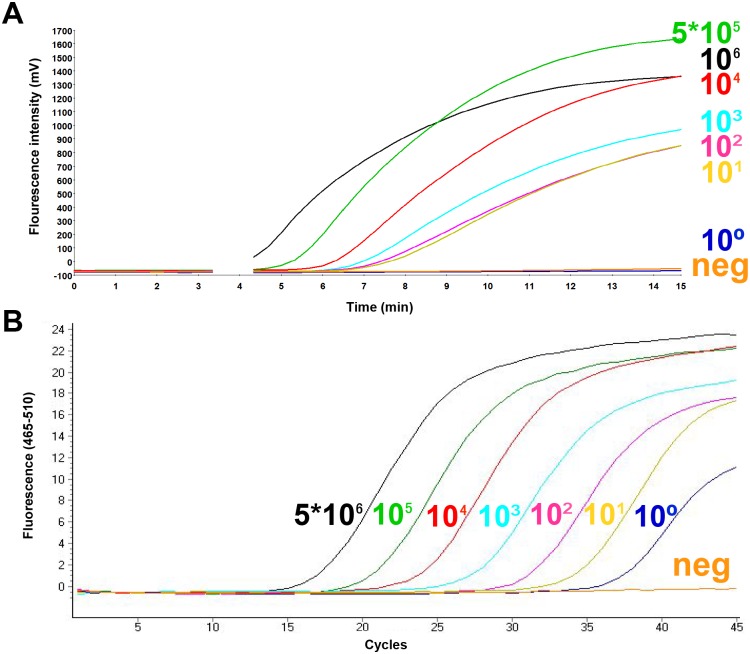
Amplification curves in MAP RPA (A) and MAP real-time PCR (B) assays applying serial dilutions (5x10^6^-1) of the molecular plasmid DNA standard. Analytical sensitivity of RPA and real-time PCR assays were 50 and 5 molecules/reaction, respectively. No fluorescent signals were detected in RPA after three minutes because the strip was taken out for mixing.

**Fig 2:**
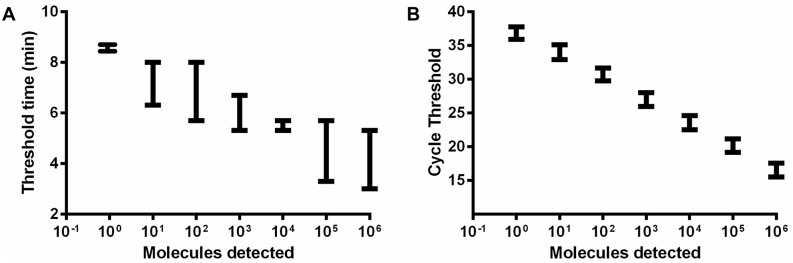
Reproducibility of the RPA (A) and real-time PCR (B) assays using data set of eight runs of serial dilution of the MAP plasmid molecular standard in PRISM. RPA assay produced results between 2 to 10 minutes. In RPA 5x10^6^-10^2^ DNA molecules were detected in 8 out of 8 runs; 50, 7/8 and 5, 1/8 by the RPA assay. The error bars represent the range. The real-time PCR produced more linear results, due to the regular cycle format of the PCR, while there is no strict separation of the amplification cycles in the isothermal RPA technology.

**Fig 3:**
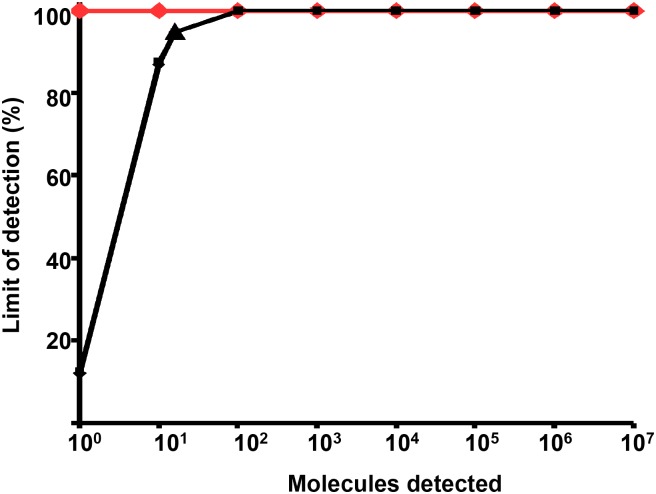
Probit analylsis results for the MAP RPA (black) and real-time PCR (orange) assays using STATISTICA. Data sets of eight RPA and real-time PCR assay runs as showed in Fig 1 was used. The limit of detection in RPA and real-time PCR at 95% probability were 16 and one DNA molecules of the molecule plasmid DNA standard, respectively.

**Fig 4:**
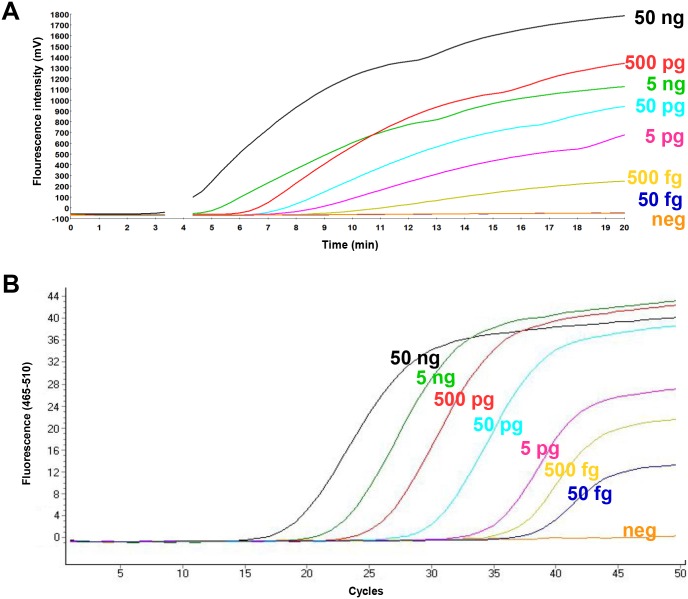
Amplification curves in RPA (A) and real-time PCR (B) using the genomic MAP DNA standard isolated from MAP (ATCC 19698). The amount of the DNA in each dilution was calculated using NanoDrop. RPA detected down to 500 fg DNA/reaction, while real-time PCR identified 50 fg DNA/reaction.

To determine the analytical sensitivity, a dilution range of the molecular DNA MAP standard was tested in RPA using the primer-probe combinations for three different regions in the IS900 gene sequence of the MAP (S1 Fig). Both RPA1 and RPA2 assays did not produce exponential amplification curves. Start points of the fluorescence signal for 5x10^5^ standard DNA were eight and seven minutes, respectively (S2 Fig). In contrast, for the primer combination FP1+RP2+P3 from RPA3 assay, the start point was between four and five minutes and the detected concentration of molecular plasmid standard was down to 50 DNA copies (Fig 1, Table 2). This combination was used for the further validation of the RPA assay. The data set of eight replicates of the molecular DNA standards of both RPA and real-time PCR assays was used in the semi-log and probit analyses. The time needed to reach the detection limit was below ten minutes in the RPA assay and 90 minutes in the real-time PCR assay (Fig 2). In probit analysis, the limits of detection with a 95% probability were 16 and one molecules of MAP plasmid standard DNA per microliter in RPA and real-time PCR assays, respectively (Fig 3). Using the genomic DNA, the limit of detection for RPA and real-time PCR assays were 500 fg/reaction and 50 fg/reaction, respectively (Fig 4).

**Table 2:** RPA primers and exo-probe combination, yielding the highest analytical sensitivity in the MAP-RPA assay.

Name	Sequence (5´ to 3´)
Probe.RPA3	ACGCCGGTAAGGCCGACCATTACTGCATGGT**QTF**TAACGACGACGCGCA
MAP.RPA3.FP1	CGTGGACGCCGGTAAGGCCGACCATTACTGCATGG
MAP.RPA3.RP2	CGCCGCAATCAACTCCAGCAGCGCGGCCTC

QTF are sites of the quencher and fluorophore in the order quencher BHQ1-dt (Q), Tetrahydrofuran (T) and Fam-dT (F).

The MAP RPA assay was highly specific, as it did not detect the DNA of other mycobacteria and environmental bacteria listed in Table 1. In addition, twenty MAP negative faecal samples were negative in both RPA and real-time PCR. Out of 48 real-time PCR positive DNA samples, forty-three were positive for MAP detection in RPA (89.5%, S1 Table).

## Discussion

In this study, we have developed a rapid RPA test detecting the IS900 gene sequence of MAP. The limit of detection using a molecular standard was 16 DNA molecules and 500 fg by employing the genomic DNA. The latter corresponds to approximately 95 MAP genomes as one MAP genome has a weight of 5.29 fg [16].

Three regions of the IS900 gene (RPA1, RPA2, RPA3, see S1 Fig) were tested in our setup. The RPA3 superposed the RPA1 and RPA2 assays in producing an exponential amplification curve and early fluorescence signal. The GC contents of RPA1, RPA2 and RPA3 regions were 70% and 67% and 55%, respectively. The higher GC content of the target gene is able to slow down the performance of the strand exchanging proteins in the reaction [20, 21], which may explain the higher sensitivity of the RPA3 region containing lower GC content.

The clinical sensitivity of our assay was around 89.5% in comparison to real-time PCR upon using archived frozen DNA extracts. Although RPA is resistant to most PCR inhibitors [22], the presence of high amount of background DNA has a negative influence on the RPA sensitivity [23]. In contrast, false negative samples in our assay contain lower amount of total DNA than positive sample as determined by Nanodrop (S1 Table). The decrease in assay sensitivity due to low MAP DNA content in the sample was also excluded as two of the false negative samples had a high content of MAP DNA as determined by real-time PCR (CT: 26–29). In addition, the RPA assay detected four samples of high CT values (between 34 to 35).

Another point of need method named Loop-mediated isothermal amplification (LAMP) was applied to detect MAP DNA [24]. The LAMP assay turnout time was 100 minutes to detect 100 fg of MAP genome. Moreover, six primers were employed to amplify the respective MAP gene. The results readout relies on the changes in the assay turbidity, which can be recognized by the naked eye or recorded in real-time by a portable device. In contrast, our RPA assay required 15 minutes process time, utilizes two primer and implemented a probe system to increase the assay specificity.

The MAP RPA assay was developed for the rapid and accurate detection of MAP DNA. A possible use of the RPA assay in combination with a fast DNA extraction protocol will allow its application for detection of MAP directly in the field.

## Supporting Information

S1 Fig MAP RPA 1–3 primer and probe sequences. (A) MAP-RPA 1 (nucleotides 483–769; Genbank accession number: AF416985.1): Nine forward primers and eight reverse primers were tested. (B) MAP-RPA 2 (nucleotides 353–645; Genbank accession number: AF416985.1): Six forward primers and fourteen reverse primers were tested. (C) MAP-RPA 3 (nucleotides 182–405; Genbank accession number: AF416985.1): Three forward primers and three reverse primers were tested. QTF are sites of the quencher and fluorophore in the order quencher BHQ1-dt (Q), Tetrahydrofuran (T) and Fam-dT (F). RC is the reverse complementary of the original sequence used in the experiment.
(PDF)

S2 Fig Amplification curves of screening primers combinations targeting three RPA regions on the IS900 gene. A, RPA1; B, RPA2; C, RPA3. RPA3 FP1+RP2 produced the best exponential curve and a start point after 4 minutes.
(TIF)

S1 Table Results of screening MAP-positive DNA samples using both real-time PCR and RPA assays. All results are mean out of triplicate. CT is the cycle threshold and TT is the threshold time. The amount of DNA in each sample was calculated using NanoDrop.
(DOC)

## Abbreviations

BHQ1: Black Hole Quencher 1
bp: Base pair
BSA: Bovine serum albumin
CT: Cycle threshold
dATP: Desoxyadenosintriphosphat
dCTP: Desoxycytidintriphosphat
dGTP: Desoxyguanosintriphosphat
DMSO: Dimethylsulfoxid
DNA: Deoxyribonucleic acid
dTTP: Desoxythymidintriphosphat
ELISA: Enzyme-linked Immunosorbent Assay
FAM: 6-carboxyfluorescein
fg: Femto gram
Fig: Figure
FP: Forward primer
g: G-Force
H_2_O: Water
LAMP: Loop mediated isothermal amplification
MAP: *Mycobacterium avium* subsp. *paratuberculosis*
MgCL_2_: Magnesium dichloride
N/A: Not available
ng: Nano gram
PCR: Polymerase Chain Reaction
P: Probe
pg: Pico gram
RPA: Recombinase Polymerase Amplification
RP: Reverse primer
rpm: Rounds per minute
sec: Second
TT: Time threshold
μl: Micro liter

## References

[1] Manning EJ, Collins MT. Mycobacterium avium subsp. paratuberculosis: pathogen, pathogenesis and diagnosis. Rev Sci Tech. 2001;20(1):133–50. 11288509 10.20506/rst.20.1.1275

[2] Chi J, VanLeeuwen JA, Weersink A, Keefe GP. Direct production losses and treatment costs from bovine viral diarrhoea virus, bovine leukosis virus, Mycobacterium avium subspecies paratuberculosis, and Neospora caninum. Prev Vet Med. 2002;55(2):137–53. 12350317 10.1016/s0167-5877(02)00094-6

[3] Benedictus A, Mitchell RM, Linde-Widmann M, Sweeney R, Fyock T, Schukken YH, et al Transmission parameters of Mycobacterium avium subspecies paratuberculosis infections in a dairy herd going through a control program. Prev Vet Med. 2008;83(3–4):215–27. 10.1016/j.prevetmed.2007.07.008 17868937

[4] Mendoza JL, San-Pedro A, Culebras E, Cies R, Taxonera C, Lana R, et al High prevalence of viable Mycobacterium avium subspecies paratuberculosis in Crohn's disease. World J Gastroenterol. 2010;16(36):4558–63. 20857526 10.3748/wjg.v16.i36.4558 PMC2945487

[5] Knosel T, Schewe C, Petersen N, Dietel M, Petersen I. Prevalence of infectious pathogens in Crohn's disease. Pathol Res Pract. 2009;205(4):223–30. 10.1016/j.prp.2008.04.018 19186006

[6] Cheng J, Bull TJ, Dalton P, Cen S, Finlayson C, Hermon-Taylor J. Mycobacterium avium subspecies paratuberculosis in the inflamed gut tissues of patients with Crohn's disease in China and its potential relationship to the consumption of cow's milk: A preliminary study. World Journal of Microbiology & Biotechnology 2005;21:1175–9.

[7] Dalziel TK. Chronic Intestinal Enteritis. The British Medical Journal 1913;2.

[8] Angelidou E, Kostoulas P, Leontides L. Bayesian estimation of sensitivity and specificity of a commercial serum/milk ELISA against the Mycobacterium avium subsp. Paratuberculosis (MAP) antibody response for each lactation stage in Greek dairy sheep. Prev Vet Med. 2016;124:102–5. 10.1016/j.prevetmed.2015.12.011 26754926

[9] Mobius P, Hotzel H, Rassbach A, Kohler H. Comparison of 13 single-round and nested PCR assays targeting IS900, ISMav2, f57 and locus 255 for detection of Mycobacterium avium subsp. paratuberculosis. Vet Microbiol. 2008;126(4):324–33. 10.1016/j.vetmic.2007.07.016 17889455

[10] Vansnick E, De Rijk P, Vercammen F, Geysen D, Rigouts L, Portaels F. Newly developed primers for the detection of Mycobacterium avium subspecies paratuberculosis. Vet Microbiol. 2004;100(3–4):197–204. 10.1016/j.vetmic.2004.02.006 15145498

[11] Plain KM, Marsh IB, Waldron AM, Galea F, Whittington AM, Saunders VF, et al High-throughput direct fecal PCR assay for detection of Mycobacterium avium subsp. paratuberculosis in sheep and cattle. J Clin Microbiol. 2014;52(3):745–57. 10.1128/JCM.03233-13 24352996 PMC3957769

[12] Lutz S, Weber P, Focke M, Faltin B, Hoffmann J, Muller C, et al Microfluidic lab-on-a-foil for nucleic acid analysis based on isothermal recombinase polymerase amplification (RPA). Lab Chip. 2010;10(7):887–93. 10.1039/b921140c 20300675

[13] Boyle DS, McNerney R, Teng Low H, Leader BT, Perez-Osorio AC, Meyer JC, et al Rapid detection of Mycobacterium tuberculosis by recombinase polymerase amplification. PloS one. 2014;9(8):e103091 10.1371/journal.pone.0103091 25118698 PMC4138011

[14] Daher RK, Stewart G, Boissinot M, Bergeron MG. Isothermal recombinase polymerase amplification assay applied to the detection of group B streptococci in vaginal/anal samples. Clin Chem. 2014;60(4):660–6. 10.1373/clinchem.2013.213504 24463560

[15] Ren H, Yang M, Zhang G, Liu S, Wang X, Ke Y, et al Development of a rapid recombinase polymerase amplification assay for detection of Brucella in blood samples. Mol Cell Probes. 2016;30(2):122–4. 10.1016/j.mcp.2016.02.007 26911890

[16] Fechner K, Schafer J, Wiegel C, Ludwig J, Munster P, Sharifi AR, et al Distribution of Mycobacterium avium subsp. paratuberculosis in a Subclinical Naturally Infected German Fleckvieh Bull. Transboundary and emerging diseases. 2015. 10.1111/tbed.12459 26671341

[17] Munster P, Volkel I, Wemheuer W, Petschenka J, Wemheuer W, Steinbrunn C, et al Detection of Mycobacterium avium ssp. paratuberculosis in ileocaecal lymph nodes collected from elderly slaughter cows using a semi-nested IS900 polymerase chain reaction. Vet Microbiol. 2011;154(1–2):197–201. 10.1016/j.vetmic.2011.06.033 21775077

[18] Zhong C, Peng D, Ye W, Chai L, Qi J, Yu Z, et al Determination of plasmid copy number reveals the total plasmid DNA amount is greater than the chromosomal DNA amount in Bacillus thuringiensis YBT-1520. PloS one. 2011;6(1):e16025 10.1371/journal.pone.0016025 21283584 PMC3026805

[19] Weisburg WG, Barns SM, Pelletier DA, Lane DJ. 16S ribosomal DNA amplification for phylogenetic study. J Bacteriol. 1991;173(2):697–703. 1987160 10.1128/jb.173.2.697-703.1991 PMC207061

[20] Patil KN, Singh P, Muniyappa K. DNA binding, coprotease, and strand exchange activities of mycobacterial RecA proteins: implications for functional diversity among RecA nucleoprotein filaments. Biochemistry. 2011;50(2):300–11. 10.1021/bi1018013 21133394

[21] Daher R. Recombinase polymerase amplification technology: Assessment for nucleic acid-based point-of-care diagnostics. Québec, Canada: LAVAL University; 2015.

[22] Piepenburg O, Williams CH, Stemple DL, Armes NA. DNA detection using recombination proteins. PLoS Biol. 2006;4(7):e204 10.1371/journal.pbio.0040204 16756388 PMC1475771

[23] Rohrman B, Richards-Kortum R. Inhibition of recombinase polymerase amplification by background DNA: a lateral flow-based method for enriching target DNA. Anal Chem. 2015;87(3):1963–7. 10.1021/ac504365v 25560368

[24] Enosawa M, Kageyama S, Sawai K, Watanabe K, Notomi T, Onoe S, et al Use of loop-mediated isothermal amplification of the IS900 sequence for rapid detection of cultured Mycobacterium avium subsp. paratuberculosis. J Clin Microbiol. 2003;41(9):4359–65. 10.1128/JCM.41.9.4359-4365.2003 12958269 PMC193777

