# Supplementary material for: Development of a Recombinase Polymerase Amplification Assay for Rapid Detection of the Mycobacterium avium subsp. paratuberculosis
Source: PLoS One. 2016 Dec 19;11(12):e0168733. doi: 10.1371/journal.pone.0168733 (PMC5167419; doi:10.1371/journal.pone.0168733)
Supplement: S1 Fig — (A) MAP-RPA 1 (nucleotides 483–769; Genbank accession number: AF416985.1): Nine forward primers and eight reverse primers were tested. (B) MAP-RPA 2 (nucleotides 353–645; Genbank accession number: AF416985.1): Six forward primers and fourteen reverse primers were tested. (C) MAP-RPA 3 (nucleotides 182–405; Genbank accession number: AF416985.1): Three forward primers and three reverse primers were tested. QTF are sites of the quencher and fluorophore in the order quencher BHQ1-dt (Q), Tetrahydrofuran (T) and Fam-dT (F). RC is the reverse complementary of the original sequence used in the experiment. (PDF) [file pone.0168733.s001.pdf]

A

Name	Sequence
Amplicon	TATATTCCCGGGCGCACGGTCCATCACGCCGCGGGTAGTTACCGCGGCGAAGGCAAGACCGACGCCAAAGACGCTGCGATCATCGCCGATCAGGCCCGGATGCGCCACGACTTGCAGCCTCTGCGCGCCGGCGATGACATCGCAGTCGAGCTGCGCATCCTGACCAGCCGACGTTCCGATCTGGTGGCTGATCGGACCCGGGCGATCGAACCGAATGCGCGCCCAGCTGCTGGAATACTTTCGGCGCTGGAACGCGCCTTCGACTACAACAAGAGCCGTGCCGCGCTG
Probe.RPA	GATCAGGCCCGGATGCGCCACGACTTGCAGCCQTFGCGCGCCGGCGATGA
Forward.1	GACGCTGCGATCATCGCCGATCAGGCCCGGATG
Forward.2	TAGTTACCGCGGCGAAGGCAAGACGCA
Forward.3	ATATTCCCGGGCGCACGGTCCATCACGCCGC
Forward.4	AGGCAAGACCGACGCCAAAGACGCTGCGATCATC
Forward.5	CGGGCGCACGGTCCATCACGCCGCGGGTAGTTA
Forward.6	TCGCCGATCAGGCCCGGATGCGCCACGACTTGCA
Forward.7	ACCGCGGCGAAGGCAAGACCGACGCCAAAGACGCT
Forward.8	AGACCGACGCCAAAGACGCTGCGATCATCGC
Forward.RP6rc	CGCCTTCGACTACAACAAGAGCCGTGCCGCGC
Reverse.1rc	CATCGCAGTCGAGCTGCGCATCCTGACCAG
Reverse.2rc	CGACGTTCCGATCTGGTGGCTGATCGGAC
Reverse.3rc	CGATCGAACCGAATGCGCGCCCAGCTGCTGGA
Reverse.4rc	GTCGAGCTGCGCATCCTGACCAGCCGACGTTC
Reverse.5rc	ACGTTCCGATCTGGTGGCTGATCGGACCCGGGCGA
Reverse.6rc	CGCCTTCGACTACAACAAGAGCCGTGCCGCGC
Reverse.7rc	ACGTTCCGATCTGGTGGCTGATCGGAC
Reverse.RP4rc.rc	AGGCAAGACCGACGCCAAAGACGCTGCGATCATC

B

Name	Sequence
Amplicon	CTGCTGGAGTTGATTGCGGCGGTGACGACGTTGGCCGATGGAGGCGAGGTCACGTGGGCGATCGACCTCAACGCCGGCGGCGCCGCGTTGCTGATCGCCTTGCTCATCGCTGCCGGGCAGCGGCTGCTTTATATTCCCGGGCGCACGGTCCATCACGCCGCGGGTAGTTACCGCGGCGAAGGCAAGACCGACGCCAAAGACGCTGCGATCATCGCCGATCAGGCCCGGATGCGCCACGACTTGCAGCCTCTGCGCGCCGGCGATGACATCGCAGTCGAGCTGCGCATCCTGAC
Probe.RPA2rc	ACGCCGCGGGTAGTTACCGCGGCGAAGGCAQTFCCGACGCCAAAGACG
RPA2.FP1	GCTGCTTTATATTCCCGGGCGCACGGTCCAT
RPA2.FP2	TCATCGCTGCCGGGCAGCGGCTGCTTTATATT
RPA2.FP3	TGCTGGAGTTGATTGCGGCGGTGACGACGTT
RPA2.FP4	GCAGCGGCTGCTTTATATTCCCGGGCGCACGGTCCATC
RPA2.FP5	GCTGCTTTATATTCCCGGGCGCAC
MAP.RPA2.RP1rc.rc	CTGCGATCATCGCCGATCAGGCCCGGAT
RPA2.RP1rc	AGACGCTGCGATCATCGCCGATCAGGCCCGGAT
RPA2.RP2rc	CGACTTGCAGCCTCTGCGCGCCGGCGATGACAT
RPA2.RP3rc	ATGACATCGCAGTCGAGCTGCGCATCCTGA
RPA2.RP4rc	GACTTGCAGCCTCTGCGCGCCGGCGATGACATCGC
RPA2.RP5rc	AGCCTCTGCGCGCCGGCGATGACAT
RPA2.FP4rc.rc	GCGGCTGCTTTATATTCCCGGGCGCACGGTCCATC
RPA2.RP7rc	TGCGATCATCGCCGATCAGGCCCGGAT
RPA2.RP8rc	ACGCCAAAGACGCTGCGATCATCGCCGATC
RPA2.RP9rc	CTGCGATCATCGCCGATCAGGCCCGGATGC
RPA2.RP10rc	ATCATCGCCGATCAGGCCCGGATGCGCCACGACTT
RPA2.RP11rc	CACGACTTGCAGCCTCTGCGCGCCGGCGATGACA
RPA2.RP12rc	ATCAGGCCCGGATGCGCCACGACTTGCAGCCT
RPA2.RP13rc	AGACGCTGCGATCATCGCCGATCAGGCCC
RPA2.RP14rc	CACGACTTGCAGCCTCTGCGCGC

C

Name	Sequence
Amplicon	GTCGGCGTGGTCGTCTGCTGGGTTGATCTGGACAATGACGGTTACGGAGGTGGTTGTGGCACAACCTGTCTGGGCGGGCGTGGACGCCGGTAAGGCCGACCATTACTGCATGGTTATTAACGACGACGCGCAGCGATTGCTCTCGCAGCGGGTGGCCAACGACGAGGCCGCGCTGCTGGAGTTGATTGCGGCGGTGACGACGTTGGCCGATGGAGGCGAGGTCAC
Probe.RPA3	ACGCCGGTAAGGCCGACCATTACTGCATGGTQTFTAACGACGACGCGCA
MAP.RPA3.FP1	CGTGGACGCCGGTAAGGCCGACCATTACTGCATGG
MAP.RPA3.FP2	GACGGTTACGGAGGTGGTTGTGGCACAACCTGTCT
MAP.RPA3.FP3	TCGGCGTGGTCGTCTGCTGGGTTGATCTGGACAAT
MAP.RPA3.RP1rc	CGATTGCTCTCGCAGCGGGTGGCCAACGAC
MAP.RPA3.RP2rc	GAGGCCGCGCTGCTGGAGTTGATTGCGGCG
MAP.RPA3.RP3rc	TGACGACGTTGGCCGATGGAGGCGAGGTCA

S1 Fig. MAP RPA 1-3 primer and probe sequences. (A) MAP-RPA 1 (nucleotides 483-769; Genbank accession number: AF416985.1): Nine forward primers and eight reverse primers were tested. (B) MAP-RPA 2 (nucleotides 353-645; Genbank accession number: AF416985.1): Six forward primers and fourteen reverse primers were tested. (C) MAP-RPA 3 (nucleotides 182-405; Genbank accession number: AF416985.1): Three forward primers and three reverse primers were tested. QTF are sites of the quencher and fluorophore in the order quencher BHQ1-dt (Q), Tetrahydrofuran (T) and Fam-dT (F). RC is the reverse complementary of the original sequence used in the experiment.
