# Supplementary figures and images for: Development of a Recombinase Polymerase Amplification Assay for Rapid Detection of the Mycobacterium avium subsp. paratuberculosis
Source: PLoS One. 2016 Dec 19;11(12):e0168733. doi: 10.1371/journal.pone.0168733 (PMC5167419; doi:10.1371/journal.pone.0168733)

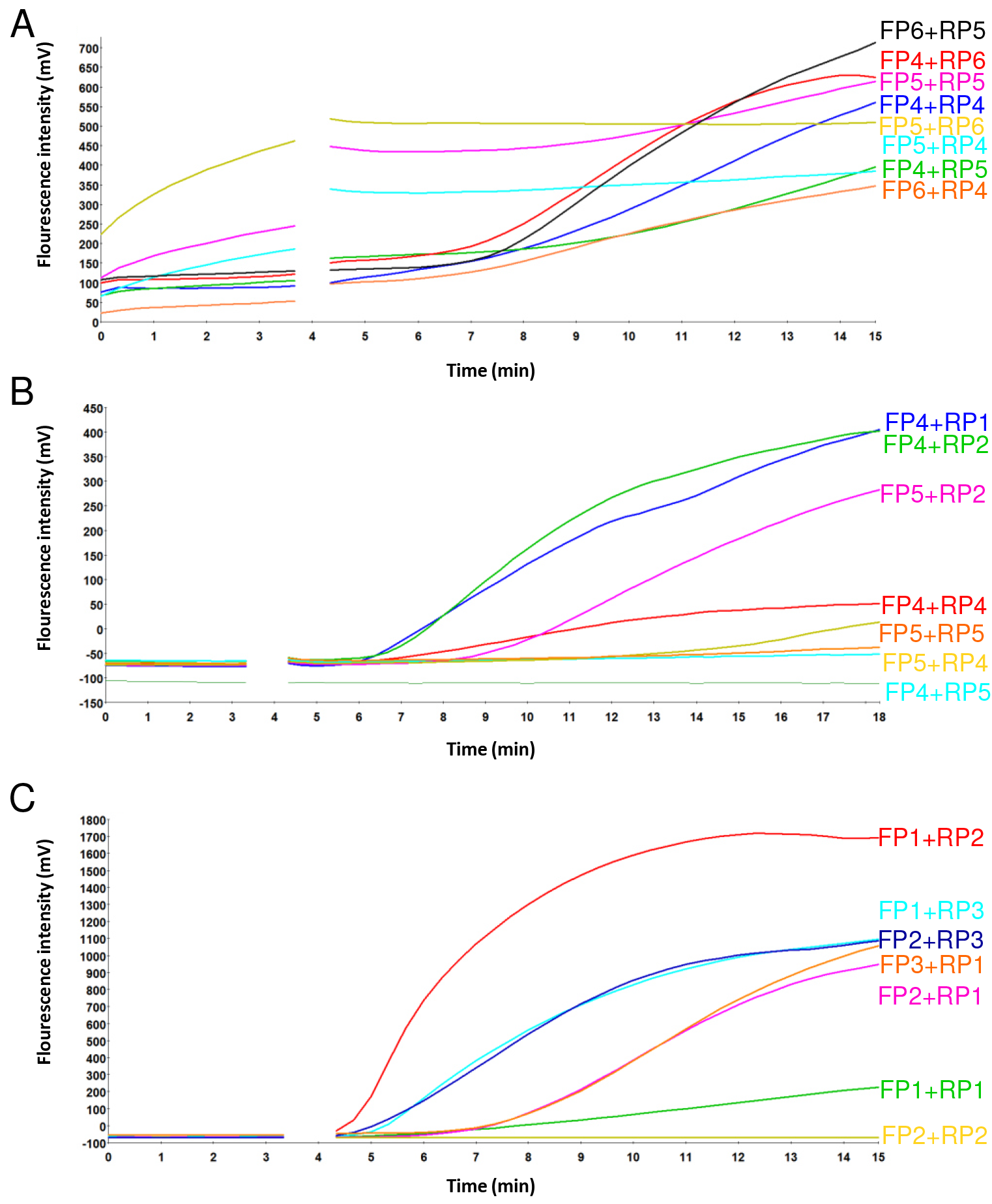

Supplement: S2 Fig — A, RPA1; B, RPA2; C, RPA3. RPA3 FP1+RP2 produced the best exponential curve and a start point after 4 minutes. (TIF) [file pone.0168733.s002.tif]
