# Supplementary material for: Development of a Recombinase Polymerase Amplification Assay for Rapid Detection of the Mycobacterium avium subsp. paratuberculosis
Source: PLoS One. 2016 Dec 19;11(12):e0168733. doi: 10.1371/journal.pone.0168733 (PMC5167419; doi:10.1371/journal.pone.0168733)
Supplement: S1 Table — All results are mean out of triplicate. CT is the cycle threshold and TT is the threshold time. The amount of DNA in each sample was calculated using NanoDrop. (DOC) [file pone.0168733.s003.doc]

**S1 Table.** Results of screening MAP-positive DNA samples using both real-time PCR and RPA assays. All results are mean out of triplicate. CT is the cycle threshold and TT is the threshold time. The amount of DNA in each sample was calculated using NanoDrop.

| **Sample ID** | **Matrix** | **Real time**  **PCR (CT)** | **RPA (TT)** | **NanoDrop (ng/µl)** |
| --- | --- | --- | --- | --- |
| **1** | feces | 30.74 | 6 | 15,11 |
| **2** | blood | 32.81 | 10,5 | 40,34 |
| **4** | feces | 32.24 | 6,5 | 11,22 |
| **5** | feces | 30,00 | 6 | 21,95 |
| **6** | blood | 32,25 | 6 | 3,92 |
| **7** | sperm | 34,81 | neg | 33,43 |
| **8** | sperm | 30,04 | 8.0 | 52,46 |
| **9** | feces | 30,81 | 8.0 | 25,38 |
| **10** | blood | 34,13 | 9.5 | 1,52 |
| **11** | blood | 30,12 | 7.0 | 13,48 |
| **12** | sperm | 29,92 | 8.0 | 19,12 |
| **13** | sperm | 32,38 | 7.25 | 53,73 |
| **14** | blood | 36,85 | neg | 12,49 |
| **15** | blood | 32,48 | 7.5 | 10,49 |
| **16** | sperm | 34,88 | 11.5 | 7,23 |
| **17** | sperm | 35,02 | 10.6 | 14,68 |
| **18** | feces | 33,42 | 7.0 | 98,92 |
| **20** | blood | 33,24 | 6.0 | 25,28 |
| **21** | blood | 29,93 | 7,5 | 33,95 |
| **22** | blood | 28,92 | 5.5 | 6,03 |
| **23** | blood | 29,28 | 6.0 | 0,64 |
| **24** | blood | 28,92 | 6.5 | 1,99 |
| **25** | sperm | 29,40 | 10.0 | 176,52 |
| **26** | sperm | 28,92 | 8.0 | 148,90 |
| **27** | blood | 31,52 | 6.5 | 7,68 |
| **28** | sperm | 31,77 | 5.35 | 186,96 |
| **29** | sperm | 32,05 | 4.65 | 175,92 |
| **30** | sperm | 30,70 | 12.0 | 147,51 |
| **31** | sperm | 29,90 | neg | 149,33 |
| **32** | feces | 32,83 | 9.25 | 78,98 |
| **33** | sperm | 26,93 | neg | 183,44 |
| **34** | sperm | 27,33 | 3.5 | 511,83 |
| **35** | feces | 22,56 | 14.25 | 92,72 |
| **36** | feces | 22,94 | 5.5 | 62,11 |
| **37** | feces | 19,03 | 4.75 | 75,88 |
| **38** | feces | 23,55 | 5.0 | 56,11 |
| **39** | blood | 20,58 | 11.0 | 11,53 |
| **40** | blood | 24,41 | 11.0 | 12,52 |
| **41** | tissue goat | 26,77 | 7.25 | 299,18 |
| **42** | tissue goat | 22,65 | 3.5 | 235,80 |
| **43** | tissue goat | 31,73 | 14,50 | 98,62 |
| **44** | tissue goat | 33,91 | 8,00 | 79,57 |
| **45** | sperm | 32,63 | 9.65 | 414,81 |
| **46** | sperm | 32,16 | 7.5 | 385,90 |
| **47** | sperm | 32,01 | neg | 286,59 |
| **48** | sperm | 32,14 | 10,00 | 250,48 |
| **49** | feces | 35,26 | 6,00 | 130,73 |
| **50** | feces | 27,05 | 7,00 | 63,70 |
